# SEER-based survival nomogram for salivary acinar cell carcinoma using clinical and surgical factors

**DOI:** 10.1097/MD.0000000000047918

**Published:** 2026-03-13

**Authors:** Xuying Zheng, Junbing He, Yufu He, Yuling Xu, Yao Lin

**Affiliations:** a The Department of Stomatology, Jieyang People’s Hospital (Jieyang Affiliated Hospital of Sun Yat-sen University), Guangdong, China; b Jieyang Medical Research Center, Jieyang People’s Hospital (Jieyang Affiliated Hospital of Sun Yat-sen University), Guangdong, China.

**Keywords:** acinar cell carcinoma, nomogram, overall survival, prognostic factor, salivary gland

## Abstract

Acinar cell carcinoma (AcCC) has a certain risk of recurrence, metastasis or even death. This study aimed to explore the relationships between clinicopathological characteristics and survival in AcCC patients, and a nomogram model was developed and validated for predicting overall survival (OS). AcCC patients were identified from the Surveillance, Epidemiology, and End Results Program. An external validation was conducted using an independent cohort from our hospital. Independent prognostic factors for OS were determined using univariate/multivariate Cox regression analyses, and a nomogram was created to predict survival. The model was validated using various methods, including calibration curves, receiver operating characteristic curves, and concordance indexes. A total of 1306 patients (916 in the training set and 390 in the validation set) with AcCC were enrolled. The results of multivariate Cox regression analysis revealed that age, sex, advanced T stage, N stage, M stage, and the type of surgery were independent prognostic factors for OS. The established nomograms incorporating the clinical factors and surgery type had robust and accurate performance according to the concordance indexes (0.824) and area under the curve values of 0.864 and 0.829, respectively, in predicting 3-year survival and 5-year survival in the training set. Receiver operating characteristic curve also showed better prognostic prediction performance for OS in the internal and external validation group. Moreover, the calibration curves exhibited excellent agreement between the actual observations and nomogram predictions. The OS of high-risk patients exhibited worse than that of low-risk patients in Kaplan–Meier survival analysis. A nomogram based on clinical features and surgery was developed for the first time and validated to predict personalized 3- and 5-year OS in AcCC patients. It helps clinicians predict survival and obtain prognostic information. Integrating this nomogram into clinical practice could improve decision-making, optimize therapy, and enhance patient outcomes.

## 1. Introduction

Acinic cell carcinoma (AcCC) is a rare low-grade epithelial malignancy in which at least some of the cells that constitute the tumor demonstrate serous acinar cell differentiation, which is characterized by cytoplasmic zymogen secretory granules.^[1]^ Controversy about the malignant nature of AcCC has existed in the past. Earlier research classified it as an “acinic cell tumor” or benign “adenoma,” as the behavior of this tumor was rather indolent.^[2]^ The majority of these tumors have well-circumscribed and slow-growing characteristics. However, its potential for recurrence, metastasis or even death is now being explored more thoroughly.^[3,4]^ In its malignant form, AcCC grows rapidly and exhibits aggressive features, with adhesion of surrounding skin or muscle tissue and invasion of adjacent nerves, frequently causing dysfunction. Currently, AcCC is more likely to be a malignant tumor with an indeterminate disease course.^[1]^

Clinically, the symptoms of patients with AcCC vary depending on the location and extent. AcCC commonly occurs in the salivary glands, among which the parotid gland is the most frequently involved site,^[1]^ followed by the submandibular and sublingual glands. Slow swelling is the most typical clinical manifestation; however, patients are asymptomatic in most instances, resulting in a late diagnosis.^[5]^ The facial nerve (FN) and salivary gland are intimately connected both functionally and anatomically, as the FN trunk exits through the stylomastoid foramen and then proceeds anteriorly through the glandular substance of the parotid gland.^[6]^ FN palsy can possibly occur due to tumorous infiltration or damage during surgery.^[7]^ Moreover, the presence of a malignant mass in the submandibular or sublingual region, along with infiltration of surrounding tissue structures, can result in symptoms such as numbness, pain, or impaired function. Invasion of the masseter muscle by the tumor may further lead to restricted mouth opening. Additionally, research has shown that the majority of relapses in patients with malignant parotid AcCC manifest as distant metastases occurring late in the disease course, representing a significant source of disease failure despite its rarity.^[8,9]^

Surgery has been the mainstay of treatment for malignant salivary gland tumors. The extent of the surgical procedure depends on the size and location of the tumor. As an important motor nerve in the maxillofacial region, the FN requires careful identification and dissection during surgery.^[10]^ FN involvement is, however, the other side of the coin, as research has shown that both clinical and histopathological evidence of perineural invasion are negative prognostic factors for patients with carcinomas of the parotid gland.^[11–14]^ It is challenging for clinicians to preserve or reconstruct the FN while performing an effective oncologic surgery. Typically, if no evidence shows that the FN is infiltrated, involved or surrounded by the tumor, all efforts should be made to preserve the FN. Instances where there was distinct clinical invasion of the nerve resulted in resection of the FN to obtain a negative margin at the gross tumor site. The management of FN has been debated for decades, with some previous researchers advocating for a more aggressive surgical approach in the early stage. Several studies have reported radical parotidectomy rates of 48% to 59%, with a considerable proportion of patients not having FN involvement.^[15]^ Importantly, however, resecting the FN inevitably leads to dysfunction of the corresponding facial muscles. AcCC is considered to have low-grade malignant potential and a favorable prognosis. There was a valid concern regarding the potential benefit of sacrificing the FN at an early stage. The primary objective of surgical therapy is to achieve complete removal of tumor tissue while minimizing harm to surrounding healthy tissue, thereby improving survival rates and quality of life. Nevertheless, further investigations are warranted regarding the various surgical approaches for managing FN involvement in patients with AcCC. Besides, the conventional TNM staging system has limitations in accurately predicting prognosis in despite of widespread use.^[16]^ While it provides crucial anatomical information regarding tumor size and extent, it fails to consider clinical and surgical factors, as well as other molecular characteristics that may more effectively predict clinical outcomes in AcCC patients. To address this issue, developing a more personalized treatment regimen and providing guidance for clinical practice necessitate a comprehensive analysis of risk factors and the development of intuitive models to visually predict the correlation between these factors and the probability of survival.

Recently, nomograms have become popular for predicting the survival of cancer patients. However, a nomogram incorporating the clinical characteristics and surgery type for predicting the overall survival of AcCC patients in the salivary gland is lacking. Thus, we established a nomogram for the first time, for predicting the 3-year and 5-year overall survival (OS) of AcCC patients via data from the Surveillance, Epidemiology, and End Results (SEER) database. This novel approach may offer new insights into the prognosis prediction and individualized treatment for AcCC patients.

## 2. Methods

### 2.1. Data source and participant selection

Data on patients diagnosed with AcCC were retrieved from the SEER Program (www.seer.cancer.gov) SEER*Stat Database on September 26, 2023: Incidence – SEER Research Data, 18 Registries, November 2020 Sub (2000–2018) – Linked To County Attributes – Time Dependent (1990–2018) Income/Rurality, 1969–2019 Counties, National Cancer Institute, DCCPS, Surveillance Research Program, released April 2021, on the basis of the November 2020 submission. We utilized publicly accessible data from the SEER database without patient identification in this study. Formal ethical approval and informed patient consent were not necessary for access to SEER database data, and its open access policy was included. Patients with AcCC were defined according to the International Classification of Diseases for Oncology, 3rd edition (ICD-O-3) histologic codes 8550/3, and the site-specific code (ICD-O-3/WHO 2008) “Salivary gland” was applied to classify AcCC confined to the salivary gland. All available qualified patients were enrolled unless the data were inadequate. Missing data were handled by exclusion. Any patient with missing key variables, such as age, sex, TNM stage, or surgery type, was excluded from the final analysis. The exclusion criteria were as follows: basic information on sex and age was not available, the American Joint Committee on Cancer (AJCC) 6th edition TNM staging system was incomplete or the stage was impossible to evaluate (TX, NX, MX), and the surgery type of the primary site was unknown or unclear (codes 99 and 90).

### 2.2. Regarding the selection of training and validation groups

The cases were randomly divided into a training group and a validation group in a ratio of 7:3 using a random number method. To further validate the randomness, the distribution of these factors was compared between the training and validation sets to confirm that they were not significantly different. The group for external validation included 33 patients diagnosed with salivary AcCC who received treatment at Jieyang People’s Hospital over the period from 2004 to 2024. The selection of patients was in accordance with the criteria outlined by the SEER database guidelines. The last follow-up was performed in April 2025. Approval for the study was granted by the Ethics Committee of Jieyang People’s Hospital, and informed consent was obtained.

### 2.3. Data extraction and statistical analysis

RStudio (version 4.1.1; RStudio Inc., Boston) and GraphPad Prism (version 9.0; GraphPad Software, Inc., San Diego) were used for the statistical analyses. OS was defined as the interval between diagnosis and death caused by any reason or the last follow-up. Six variables, including T stage, N stage, M stage, age, sex, and the type of primary surgery, were extracted. The cases were randomly divided into a validation set and a training set for model evaluation via RStudio, which included 30% and 70% of the dataset, respectively. The effect of each factor on prognosis was evaluated by univariate and multivariate Cox regression, and a nomogram was established via R packages (RStudio Inc., Boston), including the rms package, survival package, and regplot package. The concordance index (C-index) is used as an indicator of modeling performance, and a higher C-index equates to better outcomes (where 0.5 indicates no discrimination and 1.0 indicates perfect prediction). In addition, a receiver operating characteristic (ROC) curve was used to assess the discriminative ability of the nomogram model. The area under the ROC curve was calculated to quantify the ROC, and a larger area under the ROC curve implied better performance (where 0.5 indicates a random classifier and 1.0 indicates a perfect model). Moreover, calibration curves were generated to assess the agreement between the actual and predicted outcomes. The OS, which is the main outcome of this study, was compared between the groups with high and low AcCC risks using Kaplan–Meier curves. A *P* value of <.05 was considered statistically significant.

## 3. Results

### 3.1. Demographics and pathological characteristics

**Figure 1.**
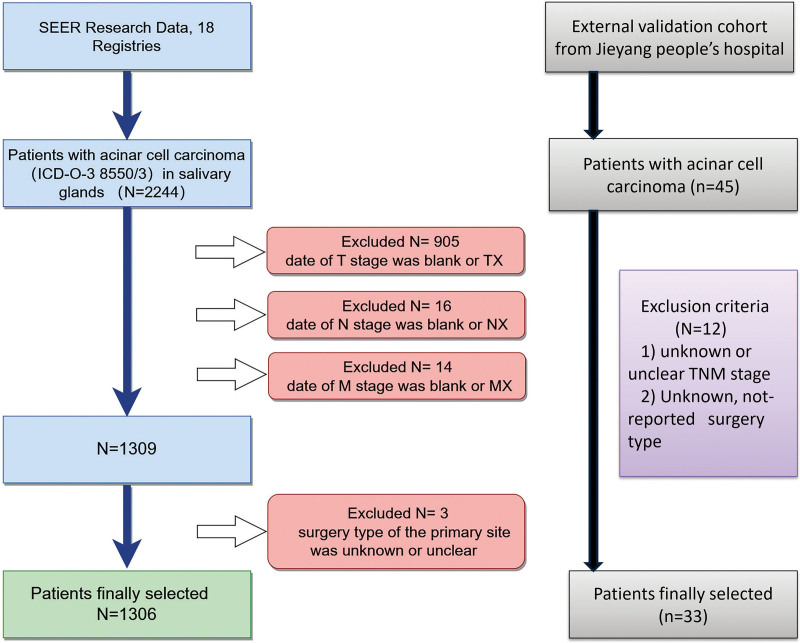
Patient selection process. SEER = Surveillance, Epidemiology, and End Results.

The patient selection process is shown in Figure 1. A total of 1306 patients were included in the present research and randomly allocated to the validation (n = 390) or training (n = 916) cohort. The demographics and pathological characteristics of the included patients are presented in Table 1. No significant difference in baseline characteristics was observed between the training and validation cohorts. The reported age at diagnosis in this database was <50 years in more than half of the patients (55.2%). Similar to a recent demographic study, 59.7% of patients were female, and 40.3% were male. The majority of patients were classified as T1 (47.2%), while a significant proportion (34.2%) were classified as T2, and 18.6% were classified as T3/T4. Only a few patients developed regional lymph node invasion and distant metastasis, among whom 4.5% were classified as N1, 3.5% were classified as N2/N3, and only 1.1% were classified as M1. Surgical resection was the main primary treatment modality, and only 2.6% of patients did not receive surgery of the primary side. The type of surgical approach included an “appropriate” salivary gland excision whereby the tumor is excised with the involved gland according to the extent of disease. The surgical types were further stratified into 4 categories according to the method of tumor resection and the treatment of the affected parotid gland and FN. The most common primary operation performed was subtotal/total salivary gland excision (SGE) with the facial nerve spared (FNSP) (SGE + FNSP, 49.6%), whereas 15.1% of patients received subtotal/total salivary gland excision with the facial nerve sacrificed (FNSA) (SGE + FNSA/radical). A total of 7.4% of AcCC patients underwent local mass removal treatment. Otherwise, subtotal/total salivary gland excision with no other specifications for the treatment of the FN (SEG no other specifications for the treatment of the FN) was performed in 25.3% of the AcCC patients.

**Table 1:** Summarized data of the cases of acinic cell carcinomas included in the present study.

Characteristics	All patients n = 1306	n (%)	Training group n = 916	n (%)	Internal validation group n = 390	n (%)	*P* value	External validation group n = 33	n (%)	*P* value
Age (yr)										
<50	585	44.8	402	43.9	183	46.9	.313	12	36.4	.392
≥50	721	55.2	514	56.1	207	53.1		21	63.6	
Sex										
Female	780	59.7	553	60.4	227	58.2	.465	18	54.5	.502
Male	526	40.3	363	39.6	163	41.8		15	45.5	
Stage_T										
T1	617	47.2	422	46.1	195	50.0	.419	12	36.4	.441
T2	446	34.2	321	35.0	125	32.1		15	45.4	
T3/T4	243	18.6	173	18.9	70	17.9		6	18.2	
Stage_N										
N0	1202	92.0	842	91.9	360	92.3	.966	28	84.9	.044
N1	58	4.5	41	4.5	17	4.4		1	3.0	
N2/N3	46	3.5	33	3.6	13	3.3		4	12.1	
Stage_M										
M0	1291	98.9	904	98.7	387	99.2	.573	32	97.0	.404
M1	15	1.1	12	1.3	3	0.8		1	3.0	
Type of surgery										
NS	34	2.6	28	3.1	6	1.5	.299	3	9.1	.028
LMRT	97	7.4	64	7.0	33	8.5		6	18.2	
SGE + FNSA/radical	197	15.1	131	14.3	66	16.9		2	6.1	
SGE + FNSP	648	49.6	462	50.4	186	47.7		15	45.4	
SGE NOS	330	25.3	231	25.2	99	25.4		7	21.2	

FNSA = facial nerve sacrificed, FNSP = facial nerve spared, LMRT = local mass removal treatment, NOS = no other specifications for the treatment of the facial nerve, NS = not receive surgery, SGE = subtotal/total salivary gland excision.

### 3.2. Prognostic factors

The results of the univariate and multiple Cox regression models for OS in the training cohort are shown in Table 2. The univariate analysis revealed that age at diagnosis over 50 years (hazard ratio (HR) = 5.765; *P* < .001), male sex (HR = 2.034; *P* < .001), advanced T stage, N stage, M stage, and no surgery of the primary side were associated with decreased OS, whereas all of the different types of surgical treatment were favorable factors (all *P* < .05). HR > 1 representing adverse prognostic factors. Furthermore, in multiple Cox regression models, age at diagnosis, advanced T stage, N stage, M stage, type of primary surgery, and sex were determined to be independent prognostic factors for OS (all *P* < .05). Notably, the surgical approach was identified as an important factor beneficial to OS in AcCC patients. Compared with those in patients who did not undergo surgery on the primary side, the HRs of mortality in the local mass removal treatment group (HR = 0.273; *P* = .002), SEG + FNSA/radical group (HR = 0.198; *P* < .001), SGE + FNSP group (HR = 0.176; *P* < .001) and SEG no other specifications for the treatment of the FN group (HR = 0.185; *P* < .001) were significantly lower. In contrast, metastasis was a strong poor prognostic factor, as the HRs were markedly greater in patients with N2/N3 stage disease (HR = 7.044; *P* < .001), N1 stage disease (HR = 3.872; *P* < .001), and M1 stage disease (HR = 3.163; *P* = .004). Moreover, patients ≥ 50 years of age also had a significantly less favorable prognosis (HR = 5.257; *P* < .001). In addition, male sex and advanced T stage were associated with a slight increase in all-cause mortality.

**Table 2:** Cox univariate and multivariate analysis on overall survival in the training cohort.

Variable	Univariable			Multivariable		
	HR	95% CI	*P* value	HR	95% CI	*P* value
Age (yr)						
<50	1			1		
≥50	5.765	(3.646, 9.117)	<.001	5.257	(3.292, 8.393)	<.001
Sex						
Female	1			1		
Male	2.034	(1.495, 2.767)	<.001	1.602	(1.155, 2.222)	<.01
Stage_T						
T1	1			1		
T2	1.504	(1.020, 2.219)	.0396	1.418	(0.953, 2.111)	.085
T3/T4	4.285	(2.763, 6.221)	<.001	2.509	(1.675, 3.758)	<.001
Stage_N						
N0	1			1		
N1	5.549	(3.513, 8.764)	<.001	3.872	(2.406, 6.231)	<.001
N2/N3	12.429	(8.074, 19.133)	<.001	7.044	(4.464, 11.114)	<.001
Stage_M						
M0	1			1		
M1	19	(9.906, 36.44)	<.001	3.163	(1.429, 7.000)	.004
Surgery type						
NS	1			1		
LMRT	0.136	(0.065, 0.287)	<.001	0.273	(0.120, 0.623)	.002
SEG + FNSA/Radical	0.198	(0.112, 0.351)	<.001	0.198	(0.104, 0.379)	<.001
SEG + FNSP	0.116	(0.069, 0.194)	<.001	0.176	(0.096, 0.325)	<.001
SEG NOS	0.126	(0.072, 0.221)	<.001	0.185	(0.097, 0.352)	<.001

95% CI = 95% confidence interval, FNSA = facial nerve sacrificed, FNSP = facial nerve spared, HR = hazard ratio, LMRT = local mass removal treatment, NOS = no other specifications for the treatment of the facial nerve, NS = not receive surgery, SGE = subtotal/total salivary gland excision.

### 3.3. Establishment and verification of the nomogram

**Figure 2.**
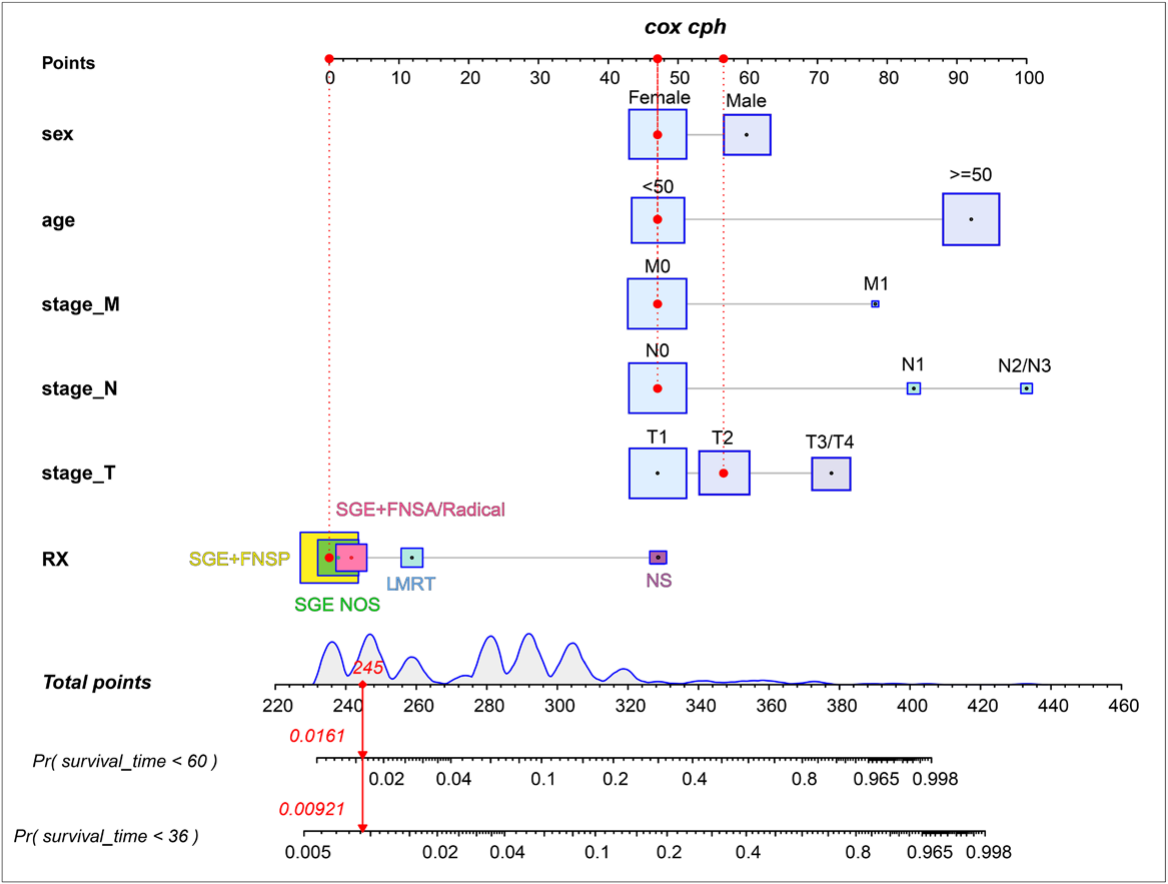
Nomogram to calculate the risk score and predict the probability of overall survival. Survival nomogram for the prediction of 3-year, and 5-year OS in AcCC patients. According to the TNM staging system of AcCC, “T” refers to the size and extent of the primary tumor, “N” indicates the degree of regional lymph node involvement, and “M” represents the presence of distant metastasis. AcCC = acinar cell carcinoma, FNSA = facial nerve sacrificed, FNSP = facial nerve spared, NOS = no other specifications for the treatment of the facial nerve, NS = not receive surgery, LMRT = local mass removal treatment, SGE = subtotal/total salivary gland excision.

**Figure 3.**
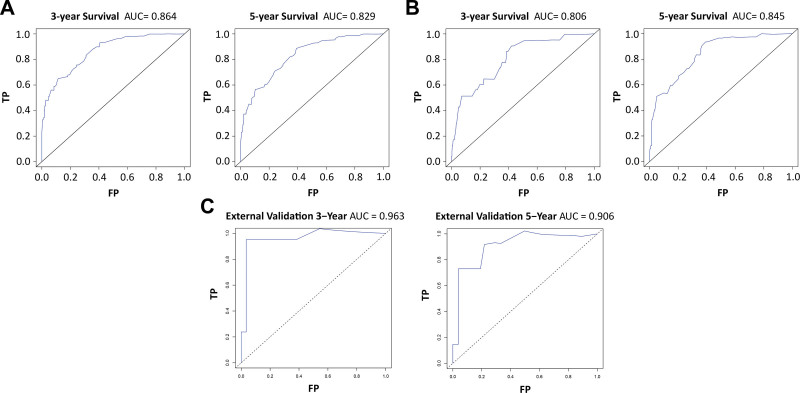
Receiver operating characteristic curve for the 3- and 5-year OS in the training group (A), internal validation group (B), and external validation group (C) of patients with AcCC. AcCC = acinar cell carcinoma, AUC = area under the curve, OS = overall survival.

**Figure 4.**
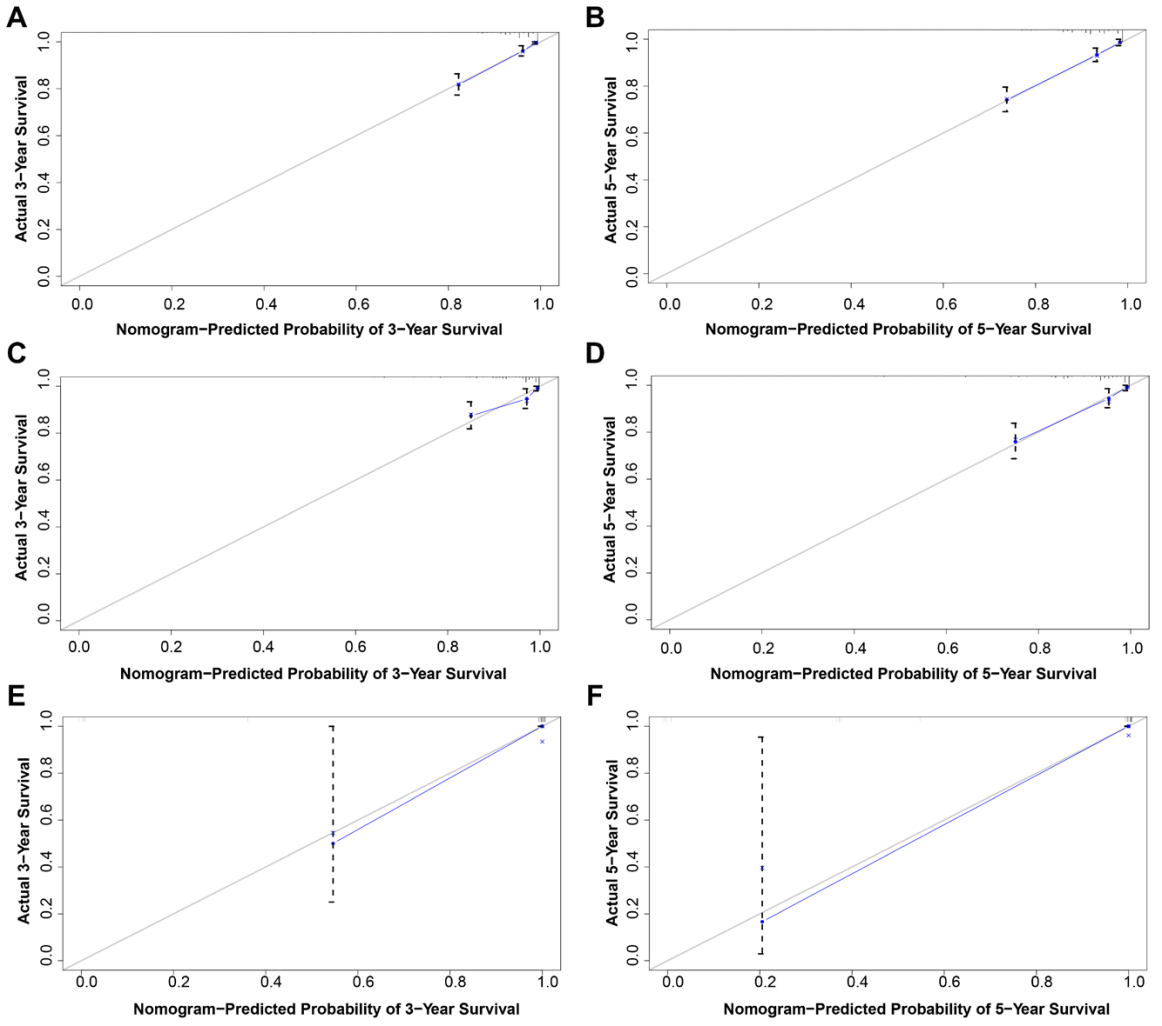
Calibration curve for 3-year-survival (A) and 5-year-survival (B) OS in the training group of patients with AcCC. Calibration curve for 3-year-survival (C) and 5-year-survival (D) OS in the internal validation group of patients with AcCC. Calibration curve for 3-year-survival (E) and 5-year-survival (F) OS in the external validation group of patients with AcCC. AcCC = acinar cell carcinoma, OS = overall survival.

In light of the multiple Cox regression analysis, a nomogram was established to predict the 3- and 5-year OS probabilities (Fig. 2). We identified the score for each variable according to the points scale at the top of the nomogram and the sum of the points for each variable. We then assessed the probability of 3- and 5-year OS on the basis of the total points scale at the bottom of the nomogram. The C-index was 0.824 (standard error = 0.017), which suggested that the nomogram was accurate for predicting OS in the training cohort. In addition, the ROC curve demonstrated good prognostic prediction performance for OS in the training group, with areas under the ROC curve (area under the curves) of 0.864 and 0.829 for 3- and 5-year OS (Fig. 3A), respectively. The ROC curve also showed better prognostic prediction performance for OS in the internal and external validation group (Fig. 3B and C). The nomogram calibration curves for OS in the training group, internal validation group and external validation group revealed good agreement between the predicted and observed survival rates at 3 and 5 years (Fig. 4).

### 3.4. Kaplan–Meier survival analysis

The analysis results highlighted the strong predictive ability of the survival nomogram. Using the 6 variables from the nomogram, we calculated a prediction score. Patients from the training cohort were separated in 2 groups according to the median cutoff value: the low-risk group and the high-risk group. As presented in Figure S1A, Supplemental Digital Content, https://links.lww.com/MD/R518, Kaplan–Meier survival analysis revealed that high-risk patients had significantly worse OS compared to low-risk patients (*P* < .001). Similarly, Kaplan–Meier survival analysis of the internal validation cohort revealed that the OS of high-risk patients exhibited significantly worse compared to those of low-risk patients (Figure S1B, Supplemental Digital Content, https://links.lww.com/MD/R518; *P* < .001). In the external validation cohort, Kaplan–Meier survival analysis showed that the OS of low-risk patients again showed a significant advantage over the high-risk group (Figure S1C, Supplemental Digital Content, https://links.lww.com/MD/R518, *P* = .001).

## 4. Discussion

Predicting survival rates is an outstanding challenge in patients with low-degree carcinoma, especially fields where there is a lack of high-level evidence to guide optimal management. Previous research on AcCC has often focused on single institution-based studies with small sample sizes, which makes statistical evaluation insufficiently powerful to identify considerable differences in survival associated with general demographic parameters.^[15,17,18]^ In our research, a total of 1306 patients with AcCC from the SEER database met the final inclusion criteria. On the basis of these large-sample data, we systematically explored and comprehensively analyzed the clinical characteristics and hazard ratios for overall survival in AcCC patients and then developed a risk stratification system for evaluating prognosis with precision and sensitivity on the basis of clinical factors and the surgery type, thus providing a meaningful reference for clinical practice.

The distribution of the clinical characteristics of AcCC patients (Table 1) in this study is consistent with the demographic spread reported in previous studies, including a predominance of females (59.7%) and older adults (aged ≥ 50 years; 55.2%).^[14,19,20]^ Typically, AcCC is considered a low-grade salivary gland carcinoma with low rates of metastasis and is generally thought to be associated with a good prognosis. Our data revealed similar results: the majority of the patients were in the T1 stage (47.2%), N0 stage (92%), and M0 stage (98.9%), whereas only a small proportion of patients developed metastasis, as 4.4% of patients were in stage N1, 3.5% of patients were in stage N2/N3, and 1.1% of patients were in stage M1. The multivariate analysis revealed that age, sex and TNM classification, as well as the surgery type, were significant predictors of OS. Age was negatively correlated with overall survival, and a large increase in the risk of death was observed in patients ≥ 50 years of age (HR = 5.257, 95% CI: 3.292–8.393). This result was consistent with a study conducted by Claudia Scherl,^[20,21]^ and this relationship has also been reflected in other salivary gland malignancies.^[22,23]^ Aging leads to a deterioration in physical fitness, and immune function may be a possible explanation for the poor prognosis of elderly individuals. Interestingly, when sex was analyzed, female patients were more prone to AcCC but had higher survival rates, whereas males had a lower morbidity but a higher risk of death (HR = 1.602, 95% CI: 1.16–2.22) (Table 2). A similar result was observed in previous studies.^[20,21]^ Research has suggested that salivary gland carcinomas are hormone dependent and that cancer risk may be influenced by excess estrogen, which may contribute to the sex disparities observed in salivary gland malignancies.^[20,24,25]^ Notably, current studies have also revealed that androgen positivity is associated with locally advanced disease and worse outcomes in patients with salivary gland carcinomas.^[26–28]^ Unfortunately, most of the relevant studies had small sample sizes and were controversial,^[29]^ and the underlying biological mechanisms remain to be further explored. In general, advanced clinical AJCC TNM staging usually indicates a worse prognosis, whereas most malignant tumors have a better prognosis in the early stages. HR = 7.04 for death among AcCC patients in the N2/N3 stage greatly increased when those in the N0 stage were as a reference. Moreover, even unilateral and single lymph node metastasis can have a significant negative impact on the risk of death (HR = 3.87). Similarly, the results of our study also suggested that patients with T3/T4 AcCC were at greater risk of death (HR = 2.509), and this finding was consistent with a previous report from Peter Moon et al.^[4]^ The study also revealed that advanced T classification was associated with a greater risk of nodal metastasis, which resulted in worse OS.

**Figure 5.**
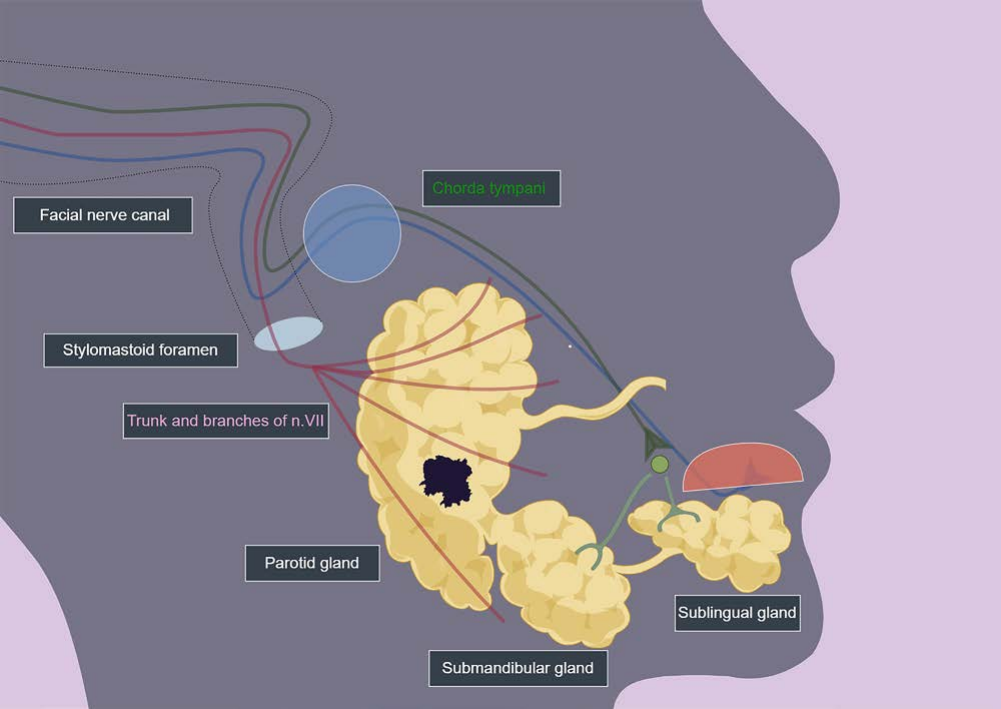
Salivary gland and facial nerve. The facial nerve course through the facial canal in the temporal bone and exit through the stylomastoid foramen, after which its main trunk divides into terminal branches in the parotid gland, and then proceeds anteriorly across the anterior edge of the parotid gland, finally distributes in the facial muscles. Chorda tympani, another branch of the facial nerve, conducts taste impulses and controls the secretion of submandibular gland and sublingual gland.

Operative treatment is the management of choice in current practice for most AcCC patients who have a resectable mass. Given that decision-making for the choice of treatment depends on the comprehensive analysis of clinical and pathological factors, the surgical procedure was included in the prognostic assessment in this study. FN, which is responsible for controlling taste perception in the tongue, facial muscle movement, and salivary gland secretion, plays a crucial role in maxillofacial surgery (Fig. 5). In our current study, we focused on the location of the removed tumor and the adjacent structures that required dissection, especially the management of the FN. In contrast to the patients who did not undergo primary surgery, the patients who received different surgery types tended to have improved OS. We observed a remarkable reduction in the risk of death among the patients who underwent subtotal/total salivary gland excision with sacrifice of the FN (HR = 0.198). Moreover, even sialadenectomy with preservation of the FN significantly decreased the HR for OS (HR = 0.176). Furthermore, the risk of death was similar for patients who underwent salivary gland resection with/without FN-sparing surgery, and the HR was slightly greater in FN-sparing/radical cases. Previous studies have shown that FN sacrifice at the time of surgery is a significant predictor of poor OS in patients with carcinoma of the parotid gland.^[4,11]^ However, nerve sacrifice is unlikely to lead to increased mortality; rather, the patients in this treatment group have a multitude of high-risk characteristics, including advanced N stage, T stage, and M stage, clinical FN palsy, and/or intraoperative gross perineural invasion, which necessitate more aggressive surgical treatment.^[4,11,30,31]^ In addition, when referring to whether more aggressive surgeries with FN sacrifice for patients in the early stage are needed, consideration should be given to balancing patient quality of life and the survival rate. Research has shown that a relatively high percentage of cases of permanent FN dysfunction are caused by sacrifice of the FN.^[11]^ Interestingly, previous research has indicated that the FN perineurium may serve as a structural barrier to tumor invasion in early-stage AcCC patients lacking high-risk histopathological features, with narrow yet negative resection margins being effective in preventing local recurrence.^[8]^ Considering that AcCC is a low-grade malignant tumor with a good prognosis, FN resection should be used with caution unless there is clear evidence of nerve infiltration during surgery. A previous study identified distinct risk factors, underscoring the importance of quantifying and incorporating these factors into clinical practice for treatment planning and prognostication in AcCC patients.

A nomogram was developed on the basis of the identified prognostic factors to accurately predict postoperative survival probability. Traditional TNM staging is a common prognostic indicator of disease outcome; however, it provides only anatomical information about the size and extent of the tumor.^[32]^ For diseases with good prognoses, hybrid prognostic models could enhance predictive efficacy, and combining clinical features enables the nomogram to be more personalized for the assessment of prognosis. Our nomogram incorporated age, sex, TNM stage, and the surgery type and exhibited accuracy and good discrimination. The C-indices of the predictive nomogram in the training group and the validation group were 0.824 and 0.820, respectively. In addition, the area under the receiver operating characteristic curve derived from our nomogram was > 0.8, indicating the satisfactory predictive performance of this model. Each variable in the nomogram was assigned different scores in a certain proportion. Therefore, in practical use, we need to obtain only the total score by adding the score that was converted from the corresponding predictive variable to the nomogram on the basis of the specific situation of each patient. Individuals with higher total scores have a greater risk of death. In this study, hypothetical scenarios were utilized to demonstrate the utilization of the nomogram calculations. The case involved a 28-year-old female patient diagnosed with AcCC in the parotid gland. The cancer stage was determined as T2N0M0 according to the AJCC TNM staging system, with no FN involvement. The patient underwent subtotal parotidectomy with preservation of the FN. The nomogram analysis revealed a 0.9% probability of survival of <3 years and a 1.6% probability of survival of <5 years. Specifically, the predicted 3- and 5-year survival probabilities of this patient were 99.1% and 98.4%, respectively, suggesting that patients with low-risk characteristics can achieve excellent prognoses even after FN-sparing surgery. Another case of an elderly male patient aged 60 years at the T4N1M0 stage with FN infiltration exhibited poor prognosis outcomes when surgery was not performed, with predicted 3- and 5-year overall survival rates of 4.8% and 0.5%, respectively. However, if this patient underwent parotidectomy excision with sacrifice of the FN, a more favorable 3-year (55.8%) and 5-year (34.9%) survival probability was forecasted. The findings from the Kaplan–Meier survival analysis indicated that the OS for high-risk patients with AcCC was notably worse than that for those low-risk patients with AcCC.

In summary, the nomogram developed in this study serves as a valuable prognostic tool for patients with AcCC, integrating clinical features and surgical variables to generate individualized risk assessments. This personalized approach enables clinicians to predict survival rates and obtain prognostic information, thereby assisting in the decisions regarding personalized treatment. For early-stage AcCC patients without FN infiltration, conservative salivary gland resection with FN preservation may be a more suitable approach. On the other hand, patients with advanced disease may benefit from more active treatment and closer monitoring of postoperative care to ensure comprehensive treatment completion. This risk stratification model empowers clinicians to allocate resources more efficiently, optimizing treatment for each patient based on their unique risk profile. Therefore, integrating the nomogram into routine clinical practice has the potential to enhance decision-making, ultimately improving both the effectiveness of therapy and the overall patient experience.^[33,34]^

There are several limitations to our research. Firstly, it was a retrospective study with some inevitable bias. Future randomized studies with large sample sizes are still needed to validate our results. Secondly, the SEER database, while a valuable resource, has limitations, including its retrospective nature and reliance on existing clinical records, which may result in incomplete or missing data, such as preoperative FN function. Regional treatment variability and the lack of standardized postoperative care data may also limit the generalizability of findings to broader patient populations. Thirdly, other factors, such as pain, drinking, smoking, and nutritional status, which may influence prognosis, are not available in the SEER database due to local data deficiencies or access restrictions. Although this nomogram demonstrated strong predictive performance in both internal and external validation, further comprehensive studies are necessary to identify more prognostic factors and improve the nomogram.

## 5. Conclusion

In the present study, a predictive nomogram for OS in patients with AcCC based on clinical features and the surgery type was generated and verified. This nomogram may be beneficial for clinicians to predict the probability of OS in AcCC patients and may be able to more precisely target patients at high risk of death, thus enabling earlier intervention, which thereby improves the quality of life and survival rate. Further randomized studies with large sample sizes are needed to validate our results.

## Author contributions

**Conceptualization:** Xuying Zheng, Yao Lin.

**Data curation:** Xuying Zheng, Junbing He, Yufu He, Yuling Xu, Yao Lin.

**Formal analysis:** Xuying Zheng, Junbing He, Yufu He, Yuling Xu.

**Funding acquisition:** Yao Lin.

**Investigation:** Xuying Zheng, Junbing He, Yufu He, Yuling Xu.

**Methodology:** Xuying Zheng, Junbing He, Yufu He, Yuling Xu.

**Project administration:** Yao Lin.

**Resources:** Yao Lin.

**Supervision:** Yao Lin.

**Validation:** Xuying Zheng, Yufu He.

**Visualization:** Xuying Zheng.

**Writing – original draft:** Xuying Zheng, Yufu He, Yuling Xu.

**Writing – review & editing:** Junbing He, Yao Lin.

## Supplementary Material
