# Supplementary material for: SEER-based survival nomogram for salivary acinar cell carcinoma using clinical and surgical factors
Source: Medicine (Baltimore). 2026 Mar 13;105(11):e47918. doi: 10.1097/MD.0000000000047918 (PMC12991712; doi:10.1097/MD.0000000000047918)
Supplement: Supplementary file 1 [file medi-105-e47918-s001.docx]

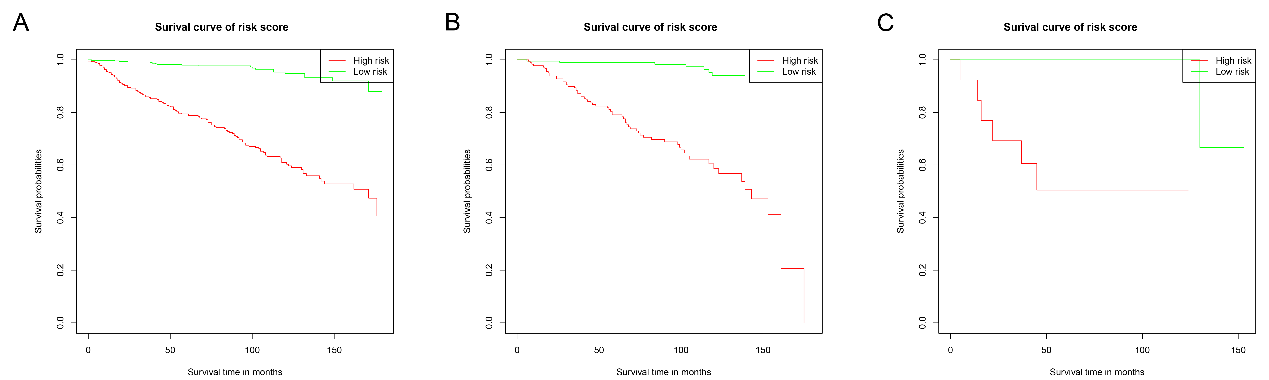


**Supplementary Figure 1**. The Kaplan-Meier survival analysis was performed to evaluate the salivary acinar cell carcinoma risk stratification system across three different groups: the training cohort (**A**), the internal validation cohort (**B**), and the external validation cohort (**C**).
